# CRISPR/Cas13a‐Based MicroRNA Detection in Tumor‐Derived Extracellular Vesicles

**DOI:** 10.1002/advs.202301766

**Published:** 2023-06-20

**Authors:** Jae‐Sang Hong, Taehwang Son, Cesar M. Castro, Hyungsoon Im

**Affiliations:** ^1^ Center for Systems Biology Massachusetts General Hospital Boston MA 02114 USA; ^2^ Cancer Center Massachusetts General Hospital Boston MA 02114 USA; ^3^ Department of Radiology Massachusetts General Hospital Boston MA 02114 USA

**Keywords:** Cas13a, CRISPR, extracellular vesicle, liposome, microRNA, ovarian cancer

## Abstract

MicroRNAs (miRNAs) in extracellular vesicles (EVs) play essential roles in cancer initiation and progression. Quantitative measurements of EV miRNAs are critical for cancer diagnosis and longitudinal monitoring. Traditional PCR‐based methods, however, require multi‐step procedures and remain as bulk analysis. Here, the authors introduce an amplification‐free and extraction‐free EV miRNA detection method using a CRISPR/Cas13a sensing system. CRISPR/Cas13a sensing components are encapsulated in liposomes and delivered them into EVs through liposome‐EV fusion. This allows for accurately quantify specific miRNA‐positive EV counts using 1 × 10^8^ EVs. The authors show that miR‐21‐5p‐positive EV counts are in the range of 2%–10% in ovarian cancer EVs, which is significantly higher than the positive EV counts from the benign cells (<0.65%). The result show an excellent correlation between bulk analysis with the gold‐standard method, RT‐qPCR. The authors also demonstrate multiplexed protein‐miRNA analysis in tumor‐derived EVs by capturing EpCAM‐positive EVs and quantifying miR‐21‐5p‐positive ones in the subpopulation, which show significantly higher counts in the plasma of cancer patients than healthy controls. The developed EV miRNA sensing system provides the specific miRNA detection method in intact EVs without RNA extraction and opens up the possibility of multiplexed single EV analysis for protein and RNA markers.

## Graphical abstract

An amplification‐free and extraction‐free extracellular vesicle (EV) miRNA detection method using CRISPR/Cas13a technology is reported. The CRISPR/Cas13a sensing components are encapsulated in liposomes and deliver them into EVs through liposome‐EV fusion. The new approach enables multiplexed protein and RNA analysis in intact EVs, quantifying specific miRNA‐positive tumor‐derived EVs in human plasma samples for more accurate cancer diagnosis.

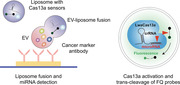

## 1 Introduction

Extracellular vesicles (EVs) are nano‐sized secretory particles originating from the cellular endosomal trafficking system.^[^ ^1^, ^2^ ^]^ EVs contain molecular cargo, including common and cell‐specific proteins, nucleic acids, and lipids, reflecting the physiological characteristics of cells of origin.^[^ ^3^ ^]^ This property makes EVs attractive as circulating biomarkers. Among various molecules contained in EVs, microRNAs (miRNAs) are a group of small non‐coding RNAs involved in post‐transcriptional gene regulation by inhibiting translation and cleavage of their target RNA transcripts. miRNAs are involved in many biological processes, such as cell development, apoptosis, cell proliferation, immune response, and tumorigenesis,^[^ ^4^ ^]^ through intercellular communications via EVs.

Recent studies have demonstrated the different roles of EV miRNAs. First, miRNAs are involved in disease initiation and progression.^[^ ^5^, ^6^ ^]^ Thus, up‐ or down‐regulation of specific miRNA can be used as a diagnostic marker for cancers,^[^ ^7^, ^8^ ^]^ cardiovascular diseases,^[^ ^9^ ^]^ lung diseases,^[^ ^10^ ^]^ and neurodegenerative diseases,^[^ ^11^ ^]^ among many others. Second, EV miRNAs play essential roles in cell‐to‐cell communications. For instance, a lymphocyte‐specific miRNA, miR‐150, released from THP1 cell‐derived EVs was delivered into the human microvascular endothelial HMEC‐1 cells, which resulted in the enhancement of cell migration by modulating c‐Myb expression.^[^ ^12^ ^]^ Therefore, accurate, quantitative measurements of EV miRNAs become critical for the clinical translation of the diagnostic biomarkers and a better understanding of EV‐medicated biological processes.

Conventional detection methods for EV miRNAs include quantitative reverse transcription polymerase chain reaction (RT‐qPCR), northern blotting, microarray, and next‐generation sequencing.^[^ ^13^, ^14^ ^]^ While these traditional methods are sensitive and provide relative quantification of target miRNAs, all require multiple steps, including EV lysis, RNA extraction, DNase treatment, cDNA synthesis, and amplification. Considering EVs’ high heterogeneity associated with different EV subtypes from almost all kinds of cells,^[^ ^15^ ^]^ EV lysis eliminates cell‐specific information in individual EVs and dilutes miRNAs in a target EV subpopulation by those from non‐target EV subpopulations. The ability to detect target miRNAs in intact EVs could significantly improve the detection accuracy and pave new ways to investigate RNA biomarkers in different EV subtypes in a multiplexed manner.

To address the technical challenges, we aim to develop a new miRNA detection technology for intact EVs. Specifically, we implemented clustered regularly interspaced short palindromic repeats (CRISPR)‐Cas system for EV RNA detection. CRISPR technology is widely used in various applications such as genome editing,^[^ ^16^, ^17^ ^]^ gene expression regulation,^[^ ^18^, ^19^ ^]^ and biosensing.^[^ ^20^, ^21^ ^]^ Recently, CRISPR‐based diagnostic technologies were developed for point‐of‐care SARS‐CoV‐2 detection.^[^ ^22^, ^23^, ^24^ ^]^ Among the Cas nucleases, we chose to use Cas13 to detect target EV miRNA molecules with CRISPR RNA (crRNA). Direct hybridization of a target RNA to crRNA activates the Cas13a's ribonuclease (RNase) activity. The activated Cas13a cleaves crRNA‐bounded target RNA (cis‐cleavage) and unbounded single‐stranded RNA molecules (trans‐cleavage).^[^ ^25^ ^]^ The latter can be used for biosensing applications along with fluorescence‐quencher (FQ) probes connected with single‐stranded RNA linkers. This approach allows us to obviate the reverse transcription step from RNA to cDNA, simplifying the EV miRNA detection.

Here, we report an amplification‐free and extraction‐free EV miRNA detection method using CRISPR/Cas13a. We incorporated CRISPR sensing components (Leptotrichia wadei/LwaCas13a, crRNA, and FQ probes) in liposomes and delivered them to EVs through EV‐liposome fusion. The fusion with lipid‐polymer nanoparticles,^[^ ^26^ ^]^ fusogenic vesicles,^[^ ^27^ ^]^ or liposomes^[^ ^24^, ^28^, ^29^ ^]^ is an efficient way to deliver sensing materials inside individual EVs. This strategy provides us with an opportunity to detect target miRNA without complex RNA extraction or amplification. After in vitro validation and assay optimization, we applied the sensing method to detect miR‐21‐5p in EVs from different ovarian cancer cell lines. miR‐21‐5p was found to be over‐expressed in ovarian cancer tissues, cells, and their EVs.^[^ ^30^ ^]^ Our detection method enabled us to quantify EV counts containing miR‐21‐5p. We found that 2%–10% of EVs from ovarian cancer cell lines showed positive signals for miR‐21‐5p, which correlates well with the bulk analysis with the gold‐standard method. We also showed that the method could be combined with the immunocapture of target‐specific EVs on gold disk arrays to measure miR‐21‐5p for EpCAM‐positive, tumor‐derived EVs. These demonstrate the specific miRNA detection in intact EVs without RNA extraction and the possibility of multiplexed single EV analysis for protein and miRNA markers.

## 2 Results and Discussions

### 2.1 Cas13a‐Mediated EV miRNA Detection System

**Figure 1:**
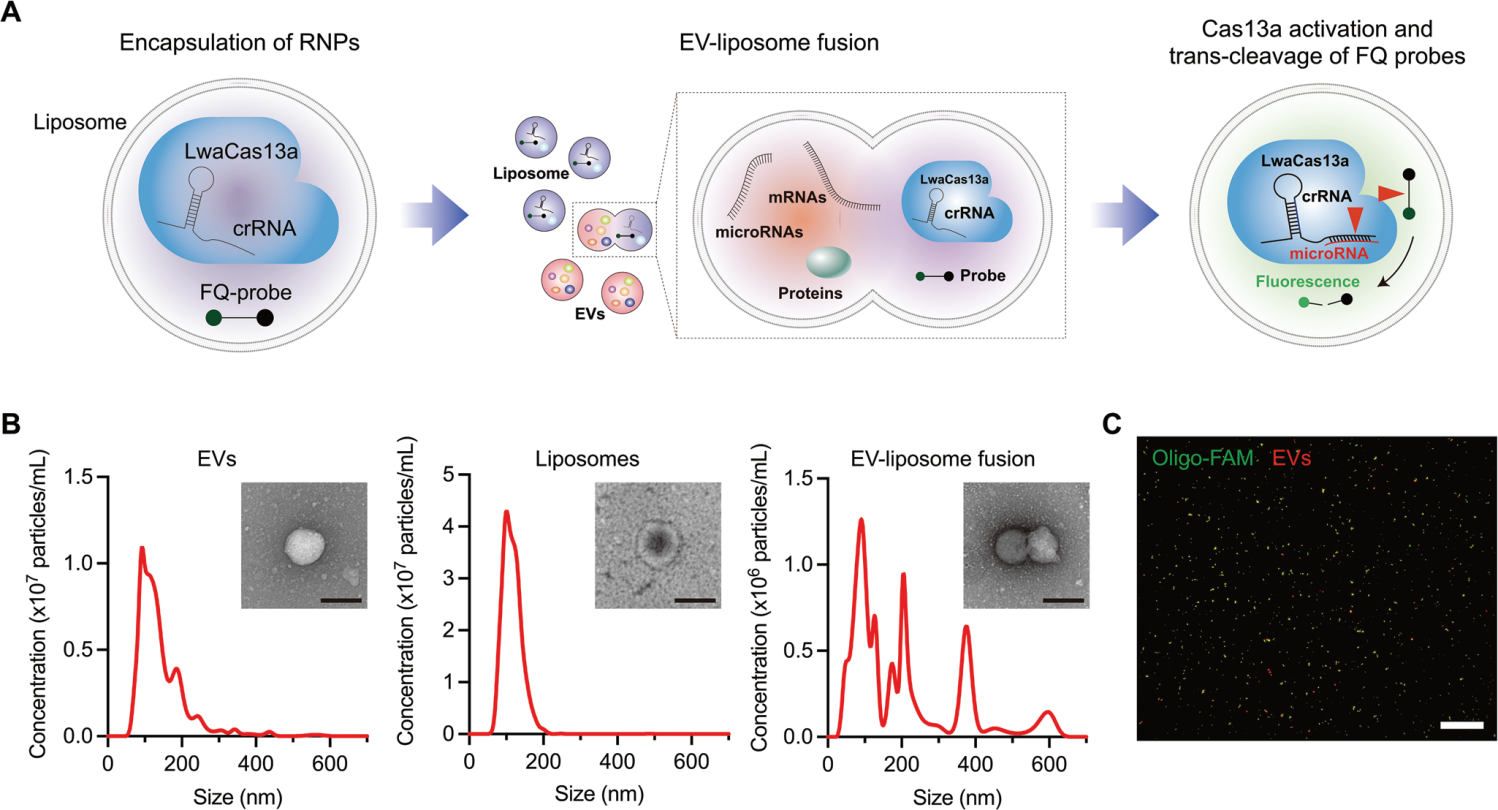
CRISPR/Cas13a‐triggered EV miRNA detection assay. A) Schematic diagram for the CRISPR/Cas13a‐based EV miRNA detection assay. Ribonucleoprotein (RNP) complexes (LwaCas13a and miRNA‐targeting crRNA) and fluorescence‐quencher (FQ) probes are encapsulated in liposomes and delivered to EVs through liposome‐EV fusion. crRNA binds to target miRNAs in EVs and activates LwaCas13a. The activated LwaCas13a cleaves FQ probes, generating green fluorescence signals inside EVs. We detect the fluorescence signals co‐localized with EV signals and quantify EV counts containing the target miRNA. B) Size distributions of EVs (*left*), liposomes (*middle*), and liposome‐EV fusion products (*right*) were measured by nanoparticle tracking analysis. The insets show transmission electron micrographs (TEMs) of an EV, liposome, and fusion particle. Scare bar, 200 nm. C) Representative fluorescence image of EV‐liposome fusion products showing excellent co–localization of fluorescently labeled EVs (red) and liposomes containing FAM‐labeled oligos (green). Scare bar, 100 µm.

Existing EV miRNA detection methods start from RNA extraction from EV lysates. This prevents us from analyzing miRNA levels in intact EVs for multiplexed analysis. To address the limitation, we developed the Cas13a‐mediated miRNA detection system for intact EVs without EV lysis. Specifically, we prepared liposomes containing LwaCas13a‐crRNA ribonucleoproteins (RNPs) and FQ probes and fused them with EVs by electrostatic interactions (**Figure** **1**A). The hybridization of target miRNAs with complementary sequence‐harboring crRNA activates LwaCas13a. The activated LwaCas13a cleaves RNA sequences in the FQ probe, generating fluorescence signals inside liposome‐fused EVs detected by a conventional fluorescence microscope. This method allows for detecting specific miRNAs in intact EVs without reverse transcription or PCR‐based gene amplification.

We first tested the detection system using EVs from the ES2 ovarian cancer cell line. We isolated EVs by size‐exclusion chromatography (please see the Methods for details). The isolated EVs showed the characteristic EV size distribution of 50–250 nm in diameter measured by nanoparticle tracking analysis (Figure 1B, *left*), which was further validated with the presence of the common EV markers, such as CD9, CD63, and CD81 by western blotting (Figure S1, Supporting Information). Next, we prepared liposomes containing RNP complexes (LwaCas13a and miRNA‐targeting crRNAs) and FQ probes using a cationic transfection reagent, Lipofectamine CRISPRMAX, which was known as the best nanocarrier for delivering Cas9 RNP complexes into target cells. The liposomes showed a similar size distribution to EVs in the range of 50–200 nm (Figure 1B, *middle*). Liposome‐EV fusion formed fusosomes with enlarged size distribution (Figure 1B, *right*). Transmission electron micrographs (TEMs) showed that EVs and liposomes displayed round‐shaped structures (Figure 1B, *left* and *middle*), and they were bound together with partial merging sites in the fusion process (Figure 1B, *right*). We calculated the liposome‐EV fusion efficiency by measuring the co‐localization of fluorescently labeled EVs and fluorescence oligomers (Figure 1C) or immunoglobulin inside liposomes (Figure S2, Supporting Information). The results show an efficient fusion rate of >80% signal co‐localization after 30 min incubation. The liposome fusion can also be applied to larger EVs, such as microvesicles and micron‐sized oncosomes (Figure S3, Supporting Information).

**Figure 2:**
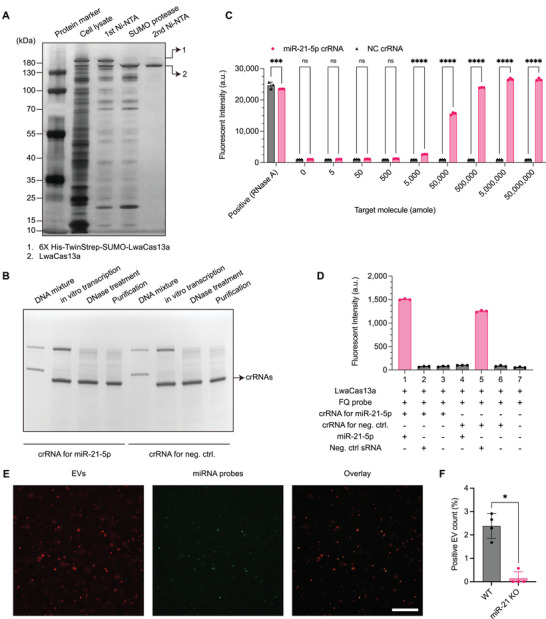
A) Purification of LwaCas13a confirmed by SDS‐PAGE followed by silver staining. B) Generation and purification of crRNA targeting for miR‐21‐5p targeting negative control (NC) crRNA with non‐complementary sequences to miR‐21‐5p. The production was assessed by denaturing PAGE and gel staining with SYBR gold. C) Activities of LwaCas13a with different concentrations of miR‐21‐5p. RNase A and NC crRNA were used as positive and negative controls, respectively. D) Specificities of the LwaCas13a‐based sensing. Positive fluorescence signals were only generated when target miRNA and complementary crRNA were present. E) Representative fluorescence images of EVs (red, *left*), LwaCas13a‐triggered miRNA signals (green, *middle*), and co‐localized signals (*right*). Scare bar, 100 µm. F) Comparison of miR‐21‐5p‐positive EV counts from the Gli261 wild‐type and miR‐21 knock‐out cell lines. * Denotes *P* < 0.05 in the Mann‐Whitney test. Bar graphs are shown as mean ± SD (*n* = 4).

We purified LwaCas13a from the bacterial expression with a LwaCas13a‐expression plasmid, followed by Ni‐NTA (nickel‐nitrilotriacetic acid) purification. Briefly, the plasmid, composed of 6XHis‐Twinstrep‐SUMO‐LwaCas13a, was transformed into the Rosetta 2(DE3) pLysS *E.coli* strain and induced by isopropylthio‐*β*‐galactoside (IPTG) treatment. Cells were lysed and purified by 1st Ni‐NTA purification to capture the N‐terminal polyhistidine residue. The eluate was then treated with polyhistidine‐tagged SUMO protease to cleave the internal SUMO cleavage sequence, upstream of the LwaCas13a sequence. Finally, the N‐terminal cleaved residue and SUMO protease were removed by 2nd Ni‐NTA purification, and unbounded LwaCas13a was collected. SDS‐polyacrylamide gel electrophoresis (PAGE) analysis by silver staining shows clear LwaCas13a product near 150 kDa size with high integrity (**Figure** **2**A; please see Experimental Section for details). We also synthesized crRNA for miR‐21‐5p and corresponding negative control by in vitro transcription, followed by DNase treatment and purification (Figure 2B; please see Experimental Section for details). The final product corresponding to crRNAs for miR‐21‐5p, and negative control were generated and clearly purified, as performed by denaturing PAGE and SYBR gold gel staining. We then investigated the trans‐cleavage activity of LwaCas13a and the cleavage specificity in vitro. In titrating miR‐21‐5p concentrations, it shows a corresponding decrease in fluorescence signals of FQ‐probes generated by the trans‐cleavage of RNA linkers by activated LwaCas13a (Figure 2C), while no signal changes were measured against non‐complementary miRNA targets (Figure 2D). This validates the specific cleavage activity of purified LwaCas13a. Additionally, we further tested the activity and specificity of the detection system in EVs from Gli261 miR‐21 knock‐out and wild‐type cell lines. We fluorescently labeled EVs by AF647 (shown in red) and analyzed their co‐localization with green fluorescence signals of FQ probes after liposome‐EV fusion (Figure 2E). The presence of miR‐21‐5p in EVs activated LwaCas13a, which cleaved FQ probes, generating green fluorescence signals. The analysis showed green fluorescence signals were co‐localized EVs’ signals well, and ≈2.4% of EVs from wild‐type Gli261 cell lines were positive for miR‐21‐5p. In contrast, the negative control EVs from the miR‐21 knock‐out Gli261 cell line (1 × 10^8^ EVs) showed a negligible background signal of 0.14% (*n* = 4, *p* < 0.05, Mann Whitney test; Figure 2F), which would be the limit of quantification.

### 2.2 Optimization of the EV miRNA Detection System

**Figure 3:**
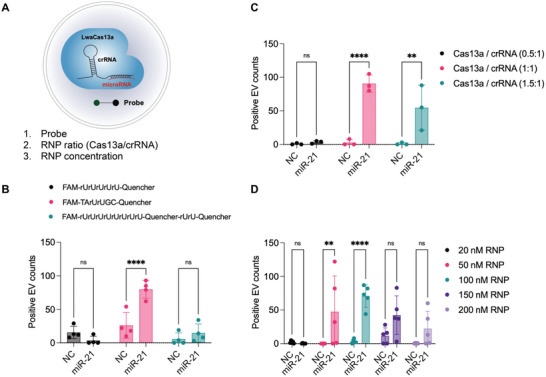
Optimization of EV miRNA detection assay. A) Three different parameters (probe sequence, RNP ratio, and RNP concentrations) are optimized for EV miRNA detection. B) Comparison of three different linkers between FAM fluorescence probes and quenchers (*n* = 4). The first has only RNA sequences (rUrUrUrUrU), the second has DNA‐RNA‐DNA hybrid sequences (TArUrUGC), and the third has double quenchers linked with RNA sequences. C) Comparison of RNP ratios between Cas13a and crRNA in 0.5:1, 1:1, and 1.5:1 (*n* = 3). D) Optimization of RNP concentration used for liposome encapsulation, ranging from 20 nm to 100 nm (*n* = 5). All bar graphs are shown as mean ± SD, and individual data were plotted as dots. ns, not significant; **p < 0.01, ****p < 0.0001 compared with NC crRNA sample, as assessed by two‐way ANOVA with Bonferroni's multiple comparisons tests.

We next optimized sensing components in liposomes (RNPs, FQ probes) for Cas13a‐based EV miRNA detection (**Figure** **3**A). We used EVs from ES2 ovarian cancer cell line for testing. As a negative control, we used liposomes with non‐complementary crRNA to measure background signals as a negative control (Figure S4, Supporting Information). We first tested three different cleavage sequences of FQ probes: 1) fluorescein amidite (FAM)‐rUrUrUrUrU‐Quencher, 2) FAM‐TArUrUGC‐Quencher, and 3) FAM‐rUrUrUrUrUrUrUrU‐Quencher‐rUrU‐Quencher. The first two probes were from previous work with the difference of RNA linker versus DNA‐RNA‐DNA linker.^[^ ^31^ ^]^ The third probe has double quenchers to test if this configuration could further decrease the background signal. The test result showed that only the probe designed with DNA‐RNA‐DNA linkers between FAM and quencher showed a significant difference between miR‐21‐5p and control samples (*p* < 0.0001, Figure 3B). The double quencher probe indeed reduced the background signals from the control sample, but the miR‐21‐positive signal also decreased, resulting in no significant difference from the control sample. Next, we tested different RNP ratios between LwaCas13a and crRNAs, and the equal molar ratio showed the most significant difference between miR‐21‐5p and control samples (Figure 3C). Furthermore, RNP concentrations of 50 nm (*p* < 0.01) and 100 nm (*p* < 0.0001) showed significant differences (Figure 3D). Taken together, we determined the optimal condition for EV miRNA detection assay with FQ probes with the DNA‐RNA‐DNA linker (TArUrUGC) and an equal amount of LwaCas13a and crRNA in 100 nm concentration. The optimized condition was then applied to the following experiments.

### 2.3 Evaluate the Abundance of EV miRNAs from Ovarian Cancer Cell Lines

**Figure 4:**
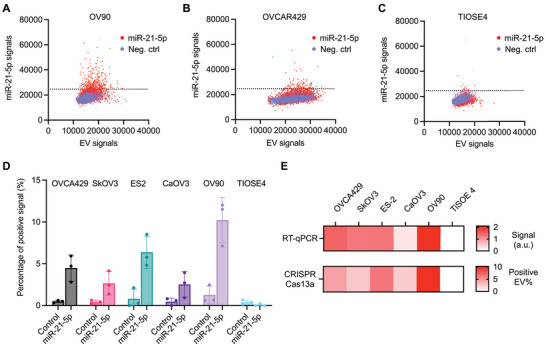
miR‐21‐5p detection in EV from ovarian cancer cell lines. A–C) Scatter plots for EVs positive for miR‐21‐5p (red) in comparison with a negative control group with non‐complementary crRNA (blue). EVs were isolated from ovarian cancer cell lines, OV90 (**A**) and OVCAR429 (**B**), as well as benign cell line, TiOSE4 (**C**). Isolated EVs were fused with liposomes containing CRISPR/Cas13a sensing components for miR‐21‐5p detection. The dotted lines indicate the intensity threshold value to determine the positivity. D) Percentages of positive signals for miR‐21‐5p from total EV counts for cell line‐derived EVs. Bar graphs are shown as mean ± SD from the three independent experiments. E) Heatmaps show the relative levels of miR‐21‐5p in EVs from different cell lines measured by RT‐qPCR (*top*) and the developed CRISPR/Cas13a detection assay (*bottom*).

We applied the CRISPR/Cas13a system to detect miR‐21‐5p in EVs from five different ovarian cancer cell lines (OVCA429, SkOV3, ES‐2, CaOV3, and OV90) and one benign cell line (TIOSE4). We first labeled isolated EVs from the cell lines, followed by fluorescent labeling using AF647 dye (please see Methods for the detailed protocol). We then applied CRISPR liposomes to 1 × 10^8^ cell line‐derived EVs and measured FAM fluorescence signals inside EVs (defined by the co‐localization with EV signals in the AF647 channel) produced from Cas13a trans‐cleavage in the presence of miR‐21‐5p (please see Figure S5, Supporting Information for fluorescence images). We used another liposome with non‐complementary crRNA as a negative control. **Figure** **4**A–C show scatter plots of EV and miR‐21‐5p signals from cell‐line derived EVs with high (OV90), moderate (OVCAR429), and low (TIOSE4) levels of miR‐21‐5p. We set the cut‐off intensities based on the negative control signals with non‐complementary crRNA, which is mean + 3 times of standard deviation. We then counted miR‐21‐5p‐positive EV percentage over the total number of EVs. The result showed that miR‐21‐5p‐positive EV counts are in the range of 2%–10% in ovarian cancer EVs (OV90, 10.22%; ES‐2, 6.37%; OVCA429, 4.48%; SkOV3, 2.66%; CaOV3, 2.53%), which is significantly higher than the positive EV counts from the benign cells (TIOSE4, 0.08%, Figure 4D).

To validate the result, we measured the relative abundance of miR‐21‐5p using the gold standard reverse transcription‐quantitative polymerase chain reaction (RT‐qPCR). Small RNAs from different cell line‐derived EV lysates were reverse transcribed, and the cDNAs were amplified with a miR‐21‐5p‐specific primer pair to measure the relative abundances of miR‐21‐5p (Figure S6, Supporting Information). Our miR‐21‐5p signals measured by our CRISPR/Cas13 system show a good correlation with the RT‐qPCT result (Pearson correlation coefficient, *r* = 0.872, *p* < 0.05, Figure 4E). Taken together, our CRISPR system for miRNA detection in EVs indicated the potential for direct detection of miRNAs in intact EVs without extra RNA isolation, reverse transcription, and target amplification.

### 2.4 miRNA Analysis in Tumor‐Derived EVs

**Figure 5:**
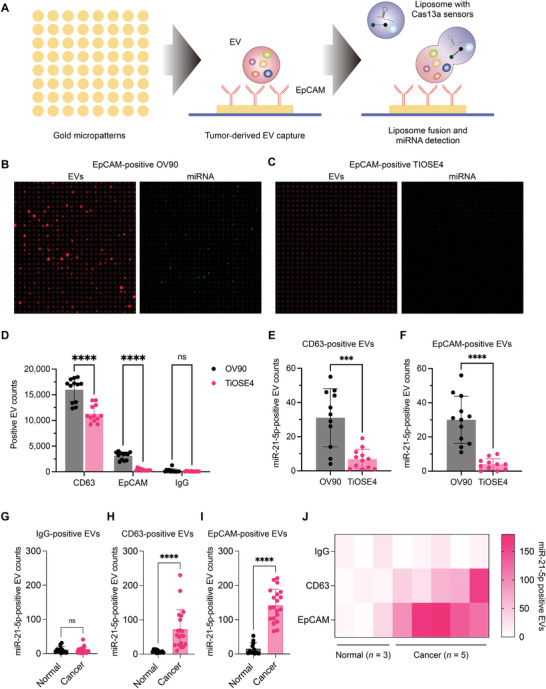
Detection of miR‐21‐5p in cancer marker expressing EVs. A) Schematic diagram for detecting miRNA signatures in EpCAM‐positive EVs. B,C) Representative images of fluorescently labeled EpCAM‐positive EVs (red*, left*) and miRNA signal (green*, right*) in OV90 EVs (B) and TiOSE4 EVs (C). D) CD63‐ and EpCAM‐positive EV counts from OV90 and TiOSE4 cells. ****p < 0.0001 compared with the OV90 and TiOSE 4, as assessed by two‐way ANOVA with Bonferroni's multiple comparisons tests. E,F) The numbers of miR‐21‐5p‐positive EVs in CD63‐ (E) or EpCAM‐positive EVs (F) from OV90 and TiOSE4 cells. ***p < 0.001; ****p < 0.0001 compared with the OV90 and TiOSE4, as assessed by two‐tailed *t*‐test. All bar graphs are shown as mean ± SD. G–I) The numbers of miR‐21‐5p‐positive EVs in IgG‐ (**G**), CD63 (**H**)‐ or EpCAM (**I**)‐positive EV populations from plasma of healthy normal donors (*n* = 3) and ovarian/mullerian cancer patients (*n* = 5). ****p < 0.0001 compared with the Normal and Cancer, as assessed by a two‐tailed *t*‐test. All bar graphs are shown as mean ± SD. J) Heatmap shows miR‐21‐5p positive EVs captured by CD63 or EpCAM antibodies. IgG isotype control was used as a negative control.

**Figure** **5**A shows the multiplexed detection strategy for tumor‐derived EVs and a miRNA marker in the subpopulation. We made gold microdisk arrays to immobilize capture antibodies using a direct physisorption method that showed ultralow non–specific binding of EVs to the gold surface.^[^ ^32^ ^]^ We designed the gold disk arrays to capture marker‐specific EVs on the designed binding areas in a grid pattern. The pattern reduces the probability of false‐positive signals from non‐specifically bound EVs (i.e., improved specificity) and simplifies image analysis. We used antibodies against CD63, a generic EV marker, and EpCAM, a cancer marker showing over expressions in ovarian cancer. We applied fluorescently labeled EVs (≈10^8^ EVs) from OV90 and TIOSE4 cell lines on the antibody‐coated gold disk arrays, followed by CRISPR‐liposome fusion for miR‐21‐5p detection in captured EVs (Figure S7, Supporting Information). The result shows that only EVs from the OV90 ovarian cancer cell line were captured by anti‐EpCAM‐coated gold disk arrays, while the binding of EVs from the TIOSE4 benign cell line was negligible, like the binding to the IgG isotype control (Figure 5B–D). For anti‐CD63‐coated gold arrays, EVs from both cell lines were captured (positive control, Figure 5D). For CD63‐ and EpCAM‐positive EVs, OV90 EVs showed a significantly higher positivity for miR‐21‐5p than TiOSE4 EVs (Figure 5E,F). However, in conventional processes by immunoprecipitation with anti‐CD63 or anti‐EpCAM followed RT‐qPCR, we could not detect miR‐21‐5p nor significant differences between OV90 and TiOSE4 EVs even if we used 20‐times higher EV amounts (≈2×10^9^ EVs; Figure S8, Supporting Information). These results indicate that the developed assay showed at least 20‐fold higher sensitivity than the conventional RNA immunoprecipitation assay for detecting miRNAs in tumor‐derived EVs.

We tested the feasibility of the assay for clinical applications. We first spiked EVs from OV90 cells into a normal plasma from a healthy donor and applied the multiplexed detection assay. After EV isolation by SEC, we captured EVs by IgG, CD63, or EpCAM antibody, followed by miR‐21‐5p detection via EV‐liposome fusion. The result shows significantly higher miR‐21‐5p‐positive EV counts among EpCAM‐positive ones in the spiked sample compared to the normal plasma (*p* < 0.0001, Figure S9, Supporting Information). We next applied the multiplexed detection to plasma samples from ovarian/mullerian cancer patients (*n* = 5) and normal healthy controls (*n* = 3, Table S2, Supporting Information). The result also showed the most significant difference of EV counts double‐positive for miR‐21‐5p and EpCAM between cancer patients and the control group (Figure 5G–J). Especially, miR‐21‐5p‐positive EV counts among the EpCAM‐positive subpopulation were 2‐fold higher than those among the CD63‐positive subpopulation.

## 3 Conclusion

We developed a new quantitative EV RNA sensing method based on liposome fusion and CRISPR/Cas13a technology. This amplification‐free, extraction‐free method obviates the need for sequential steps of EV lysis, RNA extraction, and reverse transcription, which is commonly required in conventional EV RNA analysis. More importantly, this method allows EV RNA detection in intact EVs and reports quantitative positive EV counts for specific miRNA targets. The positive EV counts we found for miR‐21‐5p in EVs from the ovarian cancer cell line were like the previously predicted amounts from the bulk analysis, one copy per 10 EVs for miR‐21.^[^ ^33^ ^]^ Still, we could quantify more precisely with specific EV counts and positive rates. Combined with the simple reaction with CRISPR/Cas13a, this leads to low background signals and a limit of quantification of 0.14%. Further study may need to validate the detection limit of a single RNA target in EVs.

Detecting EV RNAs in intact EVs for quantitative, multiplexed analysis was one of our primary goals in the method development. This is because EVs are very heterogenous with different subpopulations from various cellular origins. However, the current EV RNA analysis methods that involve EV lysis or bulk EV analysis lose the information and prevent accurate quantification within specific subpopulations. While previous similar approaches demonstrated EV miRNA detection in CD63‐positive EVs^[^ ^24^ ^]^ or all EVs,^[^ ^29^ ^]^ in our pilot study, we showed that quantification of miRNA‐positive EV counts in the tumor‐derived EV subpopulation could further increase the difference in plasma EV analysis between cancer and healthy control groups. We summarized the analytical performance of our developed system by comparing recently published EV‐miRNA detection methods (Table S3, Supporting Information).^[^ ^29^, ^34^, ^35^, ^36^, ^37^, ^38^, ^39^, ^40^, ^41^, ^42^, ^43^ ^]^ The new approach now enables quantifying EVs containing target RNA, and this new capability could be readily extended to multiplexed EV protein and RNA analysis at a single EV level.

For the delivery of sensing materials into EVs, we chose liposome‐EV fusion methods.^[^ ^26^, ^28^, ^44^ ^]^ Specifically, we used cationic liposomes based on the cationic transfection reagent Lipofectamine CRISPRMAX (Invitrogen), which was widely used for Cas9 RNP delivery into cells.^[^ ^45^, ^46^ ^]^ We showed a high fusion efficiency of >80% from signal co‐localization between fluorescent‐labeled EVs and liposomes containing fluorescent‐oligomers or IgGs, representing FQ probes or Cas13a proteins, respectively. The high fusion efficiency is crucial in the assay, as the EV RNA detection is based on CRISPR Cas13a delivery to target EVs. We envision that further optimization could be done to increase fusion efficiency. This will include testing different lipid compositions, adjusting surface charges and sizes, and applying electroporation. For RNA sensing, we chose CRISPR/Cas13a system to simplify the assay procedure while maintaining good sensitivity. Among CRISPR proteins, Cas13a (CRISPR‐class2, type VI) has been reported targeting single‐stranded RNA results in cis‐ and trans‐cleavage of RNA sequences.^[^ ^47^, ^48^, ^49^ ^]^ Therefore, it has been previously used for detecting circulating miRNAs^[^ ^50^, ^51^ ^]^ as well as SARS‐CoV‐2 detection in diagnosis.^[^ ^52^, ^53^, ^54^, ^55^ ^]^ Moreover, an activated Cas13a protein has signal amplification property of trans‐cleavage with at least 10^4^ turnovers per target.^[^ ^56^ ^]^ We found that the stability and specificity of crRNA vary by their sequences, which requires careful design and pre‐analytical validations. Combining these considerations, we developed the new EV miRNA sensing system that showed good specificity, as tested with EVs from miR‐21 knock–out cells.

With the proof‐of‐concept study, the clinical applicability for cancer diagnosis needs further clinical tests with larger cohorts. However, the current study presented the translational potential of multiplexed EV protein and RNA analysis in clinical samples. We envision that the system could be further improved toward multiplexed EV analysis. The system can be integrated with single EV analysis technologies to enable such an analysis at a single EV level.^[^ ^57^ ^]^ This new capability will significantly improve our understanding of underlying EV biology and EVs' roles as biomarkers and molecular messengers in cell‐to‐cell communications.

## 4 Experimental Section

### Bacterial Expression and Purification of LwaCas13a

LwaCas13a by bacterial expression followed by Ni‐NTA purification was purified (Figure 2A). The LwaCas13a‐expression plasmid, pC013 – Twinstrep‐SUMO‐huLwCas13a, was a gift from Feng Zhang (Addgene plasmid # 90 097; http://n2t.net/addgene:90097; RRID: Addgene_90 097).^[^ ^58^ ^]^ The plasmid was transformed into the bacterial protein expression *E.coli* strain, Rosetta 2(DE3) pLysS (Millipore Sigma) by heat shock. The single colony was inoculated and then cultured in terrific buffer at 37 °C until the value of optical density at 600 nm reached 4. Next, the transformed cells were incubated at 21 °C for 16 h after 0.5 mm IPTG induction. The cells were harvested and stored at −80 °C until protein purification. For purification of LwaCas13a, the cells were lysed in bacterial cell lysis buffer (50 mm NaH_2_PO_4_, 500 mm NaCl, 1 mg mL^−1^ lysozyme, and protease inhibitor) at 4 °C for 30 min and followed by sonication for 1 h in cold condition. The lysed bacterial proteins were collected by centrifugation with 10000 X G for 1 h at 4 °C. While collecting lysate, the NI‐NTA column was prepared. The Ni‐NTA resin (ThermoFisher Scientific) was packed into the glass Econo‐Column (BioRad) and washed twice with excess Ni‐NTA wash buffer (50 mm NaH_2_PO_4_, 500 mm NaCl, 0.2% Triton X‐100, 20 mm imidazole, and protease inhibitor). The lysate was introduced into the NI‐NTA column and incubated at 4 °C for 2 h with gentle shaking, followed by draining the flow‐through and washing the Ni‐NTA resin with Ni‐NTA wash buffer. The resin‐bounded proteins were eluted with Ni‐NTA elution buffer (50 mm NaH_2_PO_4_, 500 mm NaCl, and 250 mm imidazole). The eluate was concentrated by centrifugal filter (Millipore Sigma, 100 K MWCO) with the addition of SUMO cleavage buffer (30 mm Tris‐HCl (pH 8.0), 500 mm NaCl, and 1 mm DTT). Next, the filtrate was treated with SUMO protease (ThermoFisher Scientific) that was tagged with polyhistidine at 5’ terminus at 4 °C for overnight with gentle shaking. The new Ni‐NTA column was prepared and pre‐washed with SUMO‐NI‐NTA wash buffer (30 mm Tris‐HCl (pH 8.0), 500 mm NaCl, 20 mm imidazole, and 1 mm DTT). The SUMO protease‐cleaved product was introduced into the NI‐NTA column to remove the SUMO protease and incubated at 4 °C for 2 h with gentle shaking. The flow‐through was collected and concentrated with the centrifugal filter (100 K MWCO) with the addition of storage buffer (50 mm Tris‐HCl (pH 7.5), 600 mm NaCl, and 2 mm DTT). The protease inhibitor and 5% glycerol were added to the filtrate and stored until used at −80 °C.

### crRNA Generation

The crRNAs for miRNA detection were generated by in vitro transcription (Figure 2B). Briefly, DNA templates, which were composed of T7 promoter sequence and their complementary sequence, were annealed by controlling temperature from 95 °C to room temperature in annealing buffer (20 mm Tris‐HCl (pH 8.0), 50 mm NaCl, and 1 mm EDTA). In vitro transcription was performed by manufacturer's instruction (Promega) at 37 for 30 min, followed by DNaseI treatment for eliminating template DNAs. The transcribed RNA was then isolated by ethanol precipitation and confirmed the concentration by Qubit RNA assay kit (ThermoFisher Scientific) and quality by 15% denaturing PAGE, followed by SYBR gold staining (ThermoFisher Scientific).

### In Vitro LwaCas13a‐Mediated miRNA Detection Assay

The LwasCas13a reaction mixture was prepared with 100 nm LwaCas13a, 100 nm NC or miR‐21‐5p crRNA, 20U RNase inhibitor (Promega), 40 nm 6‐carboxyfluorescein (FAM)‐tagged quencher reporter, and indicated concentration of NC small RNA or mature miR‐21‐5p in 1X Cas13a reaction buffer (50 µL with 40 mm Tris‐HCl in pH 7.5, 60 mm NaCl, and 6 mm MgCl_2_) and incubated at 37 °C for 30 min. The fluorescent intensities were measured by a multi‐plate reader (Tecan).

### Cell Culture

The human carcinoma cell lines, including OVCA429, SkOV3, OV90, CaOV3, and ES‐2 cells, were purchased from American Type Culture Collection (ATCC). TIOSE4 was obtained from transfection of hTERT into NOSE cells maintained in 1:1 Media 199:MCDB 105 with gentamicin (25 µg mL^−1^), 15% heat‐inactivated serum, and G418 (500 µg mL^−1^).^[^ ^59^ ^]^ OVCA429, SkOV3, OV90, and TiOSE4 cells were maintained in RPMI‐1640 (Hyclone). ES‐2 and CaOV3 cells were cultured in McCoy's 5A (Gibco) and DMEM (Hyclone), respectively. All complete media were supplemented with 10% fetal bovine serum (FBS, ThermoFisher Scientific), 100 U mL^−1^ penicillin, and 100 µg mL^−1^ streptomycin (Cellgro) at 37 °C in 5% CO_2_. All cell lines were tested and confirmed that they were free of mycoplasma, as conducted with Universal Mycoplasma Detection Kit (ATCC).

### Clinical Samples

Plasma samples were obtained from ovarian cancer patients with informed consent. The protocol of the present study adhered to the Declaration of Helsinki and was approved by the Institutional Review Board of Dana Farber Cancer Institute (IRB Protocol Number: 12–238, PI: Cesar M. Castro). Blood samples were collected from patients and processed at Massachusetts General Hospital. Briefly, 10 mL peripheral blood was collected into polypropylene tubes containing sodium citrate (Vacutainer System; BD Biosciences). Whole blood samples were centrifuged at 1500 × g for 15 min at 4 °C, and plasma layers were obtained. Plasma samples were aliquoted and frozen at −80 °C until further analysis. Normal plasma samples from healthy donors were obtained from the MGH Biobank. The clinical information of patients was listed in Table S2 (Supporting Information).

### EV Isolation and Fluorescence Labeling

For EV collection from different cell lines, the cells were cultured in a complete medium until they were 80%–90% confluent. After brief PBS washing for 2 times, the cells were incubated with the basal medium for each cell line supplemented with 1% exosome‐depleted FBS (ThermoFisher Scientific), 100 U mL^−1^ penicillin, and 100 µg mL^−1^ streptomycin for 48 h. The EV isolation was conducted by size exclusion chromatography (SEC), as per the previous description.^[^ ^60^, ^61^ ^]^ Briefly, the conditioned medium was collected with a 40 µm‐sized cell strainer (Corning) and spun at 300 x g for 5 min to remove the cell debris. The supernatant was filtered through a 0.8 µm membrane filter (Millipore Sigma) and spun at 3500 x g for 30 min at 4 °C by using Centricon Plus‐70 Centrifugal Filter (MWCO = 10 kDa, Millipore Sigma). The concentrates were loaded onto the top of the SEC column, which was packed with 10 mL of Sepharose CL‐4B (GE Healthcare) in a 10 mL syringe (BD Biosciences). The fractions of 4 and 5 of 1 mL were collected and concentrated with the Amicon Ultra‐2 Centrifugal Filter (MWCO = 10 kDa, Millipore Sigma) at 3500 x g for 30 min at 4 °C. The protease and phosphatase inhibitor cocktail (ThermoFisher Scientific) were added and stored until use at −80 °C. For EV isolation from plasma samples, 1 mL of plasma samples were centrifuged at 15000 × g for 20 min at 4 °C to remove the cryoprecipitate, apoptotic bodies, and platelet and then loaded onto an SEC column and performed by same procedures as mentioned above.

EVs were labeled with AF647 dye, as previously reported.^[^ ^62^ ^]^ Briefly, 27.5 mm of Azido‐dPEG®₁₂‐TFP ester (Quanta Biodesign) and 25 mm of AFDye 647 DBCO (Click Chemistry Tools) were prepared in anhydrous DMSO (Millipore Sigma) and mixed in an equal volume followed by incubation at RT for 2 h. Next, 3 µL of EVs in PBS, 2 µL of 100 mm sodium bicarbonate (Millipore Sigma), and 0.2 µL of TFP‐AF647 were mixed and incubated at RT for 1 h. The labeled EVs were diluted with PBS in an appropriate concentration before use.

### Transmission Electron Microscope (TEM) Analysis

EVs and EV‐liposome fused particles were fixed with 2% paraformaldehyde (PFA, Electron Microscopy Sciences). These were loaded onto formvar carbon‐coated EM grids (Ted Pella) and incubated for 20 min. The grids were washed with PBS and transferred to 1% glutaraldehyde, and incubated for 5 min. The grids were then washed with distilled water. Next, the particles were stained with a mixture of 2% uranyl acetate (Electron Microscopy Sciences) and 75 mm oxalic acid (Millipore Sigma) for 1 min. The grids were dried for 5 min at room temperature after the removal of the excessive solution with filter paper. The liposomes were prepared by the same procedure without fixation. Finally, the vesicles were imaged by transmission electron microscopy (Hitachi) at 100 kV.

### Nanoparticle Tracking Analysis

The numbers and size distributions of EV, liposome, and fusion particles were analyzed by nanoparticle tracking analysis (Nanosight LM10, Malvern). Each sample was prepared by 1000‐fold dilution with PBS and manually loaded in the chamber with a 1 mL syringe. The particles were detected using a 405 nm laser module, and their Brownian motions at room temperature were captured by a CCD camera with a camera level 13 in the NTA software. Each measurement was recorded for 30 s with a 30‐frame rate per second, and each sample was measured in quadruplicate. The particle analysis was conducted with a detection threshold of 2.

### EV‐Liposome Fusion‐Based miRNA Detection Assay in Solution

The liposome complex was prepared with Lipofectamine CRISPRMAX Cas9 Transfection reagent (ThermoFisher Scientific) by a brief modification of the manufacturer's instructions. Briefly, complex A, including 100 nm LwaCas13a, 100 nm NC or miR‐21‐5p crRNA, 20U RNase inhibitor, and 40 nm FAM‐tagged quencher reporter, was prepared in 1X Cas13a reaction buffer. Additionally, complex B was prepared by gently mixing transfection reagent with 1X Cas13a reaction buffer in an equal volume of complex A. The complexes A and B were gently mixed and incubated at RT for 15 min. Next, the AF647‐labeled EVs diluted in 1X Cas13a buffer were introduced into the complex and incubated at 37 °C for 30 min. After incubation, the fusion complex was mounted on the TPFE‐printed glass slide (Electron Microscopy Sciences) and analyzed using an upright fluorescent microscope (Zeiss).

### EV‐Liposome Fusion‐Based miRNA Detection Assay

Antibody immobilization on gold microdisk arrays or plain gold was conducted, as described previously.^[^ ^32^ ^]^ Briefly, IgG isotype control (14‐4714‐82, eBioscience), CD63 (215‐820, Ancell) or EpCAM (MA5‐12436, Invitrogen) antibodies, respectively diluted by 1:50, 1:100 or 1:20 in 10 mm phosphate buffer, were treated on the surface for 1 h. After washing out the non‐bounded antibodies with PBS, 10% BSA in PBS was treated for blocking. Next, the AF647‐labeled EVs derived from cell cultures (OV90 and TiOSE4) or clinical plasma samples were incubated for 1 h. The complex for the EV‐miRNA detection system was then introduced and incubated at 37 °C for 1 h. After washing with PBST, the fluorescent signal was obtained by an upright fluorescent microscope (Zeiss).

### Western Blotting

EVs derived from ovarian cancer lines were lysed in LIPA lysis buffer (Cell Signaling Technology) containing protease inhibitor (Roche). Western blot analysis was performed as described previously.^[^ ^60^ ^]^ The nitrocellulose membrane blots were probed with anti‐CD9 (1:500 dilution, BD Biosciences), anti‐CD63 (1:500 dilution, Ancell), and anti‐CD81 (1:500 dilution, Santa Cruz Biotechnology). The chemiluminescence signal was detected with an Azure 280 imaging system (Azure Biosystems).

### Quantitative RT‐PCR Analysis

300 ng of small RNAs from 2 × 10^9^ EVs of 6 different ovarian cancer cell lines were analyzed by RT‐qPCR to evaluate the miRNA expression levels as described previously.^[^ ^63^ ^]^ Briefly, small RNAs were reverse transcribed and polyadenylated simultaneously with oligo(dT)‐linked adaptor oligomer (Table S1, Supporting Information) at sequential 37 °C for 1 h, 42 °C for 30 min, and 70 °C for 10 min. The quantitative PCR was performed with cDNAs, gene‐specific forward and universal reverse primer set, and 2X SsoAdvanced Universal SYBR Green Supermix (Bio‐Rad) by CFX Opus Real‐Time PCR Systems (Bio‐Rad). The relative miRNA expression was calculated by the 2–∆∆Ct method. U6 snRNA served as an internal control. All oligonucleotides used in RT‐qPCR were listed in Table S1 (Supporting Information).

### EV‐RNA Immunoprecipitation

EV‐RNA immunoprecipitation was performed using CD63 (215‐820, Ancell) or EpCAM (MA5‐12436, Invitrogen) antibody‐coupled to Dynabeads Protein A/G (Invitrogen) for 2 h at 4 °C in PBS. The immunoprecipitates were washed with PBS, and co‐immunoprecipitated EV small RNAs were purified using TRI Reagent (Molecular Research Center). Aliquots of EV immunoprecipitate were validated by western blotting, and the isolated small RNAs were analyzed by RT‐qPCR as described above.

### Statistical Analysis

The data were analyzed with GraphPad Prism version 9 (GraphPad Software Inc., San Diego, CA, USA). All data were displayed as mean ± standard deviation. The Mann‐Whitney unpaired *t*‐test and two‐way ANOVA with Boneferroni's multiple comparisons tests were used to compare with control sets. Statistical significance was accepted for values of *p* < 0.05.

## Conflict of Interest

H.I. is a consultant to Aikili Biosystems, Noul, and Cellkey and receives research support from Canon USA, CytoGen, Healcerion, and Noul. CMC is a consultant to Aikili Biosystems, Qiagen, Teladoc, and InfiniteMD. The other authors declare no conflict of interest.

## Author Contributions

H.I. and J.S.H. conceived the idea and designed the study. J.S.H., T.S., and C.M.C. conducted experiments. All authors analyzed the results. J.S.H. and H.I. wrote the manuscript, edited by all authors.

## Supporting information

Supporting Information

## Data Availability

The data that support the findings of this study are available from the corresponding author upon reasonable request.
