# Supplementary material for: CRISPR/Cas13a‐Based MicroRNA Detection in Tumor‐Derived Extracellular Vesicles
Source: Adv Sci (Weinh). 2023 Jun 20;10(24):2301766. doi: 10.1002/advs.202301766 (PMC10460892; doi:10.1002/advs.202301766)
Supplement: Supplementary file 1 — Supporting Information [file ADVS-10-2301766-s001.pdf]

ADVANCED SCIENCE
Open Access

Supporting Information

for *Adv. Sci.*, DOI 10.1002/advs.202301766

CRISPR/Cas13a-Based MicroRNA Detection in Tumor-Derived Extracellular Vesicles

*Jae-Sang Hong, Taehwang Son, Cesar M. Castro and Hyungsoon Im**

Supporting Information

CRISPR/Cas13a-based microRNA detection in tumor-derived extracellular vesicles

Jae-Sang Hong[1], Taehwang Son[1], Cesar M. Castro[1,2], Hyungsoon Im[1,3]*

[1] Center for Systems Biology, Massachusetts General Hospital, Boston, MA, USA
[2] Cancer Center, Massachusetts General Hospital, Boston, MA 02114, USA
[3] Department of Radiology, Massachusetts General Hospital, Boston, MA, USA

*Corresponding author:
Hyungsoon Im (im.hyungsoon@mgh.harvard.edu)

SUPPLEMENTARY FIGURES

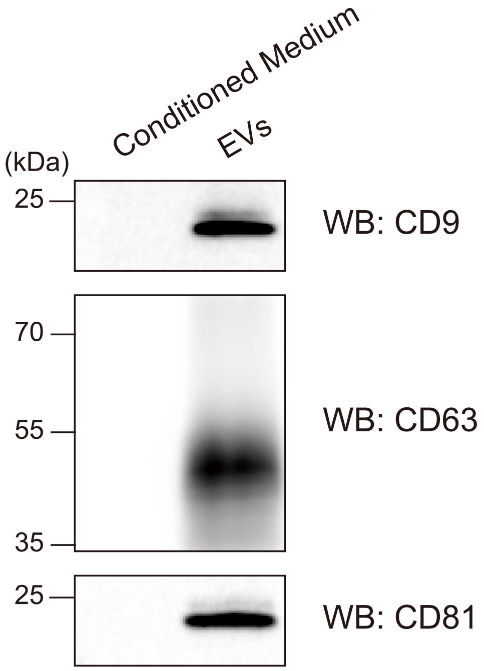

Figure S1. Western blot analysis. The tetraspanins (CD9, CD63, and CD81) were detected in EVs derived from the ES2 ovarian cancer cell line.

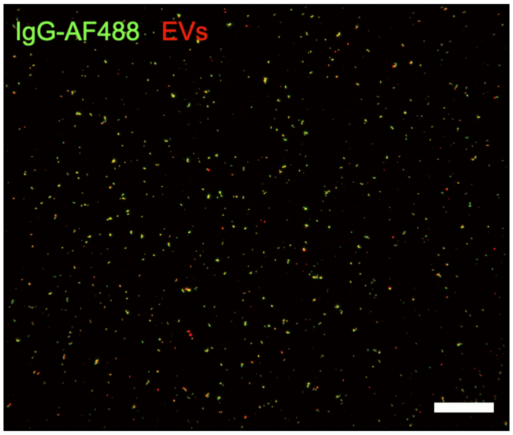

Figure S2. Representative fluorescence image of fluorescently labeled EVs (red) and cationic liposomes containing fluorescent-conjugated immunoglobulins. Scare bar, 150 µm.

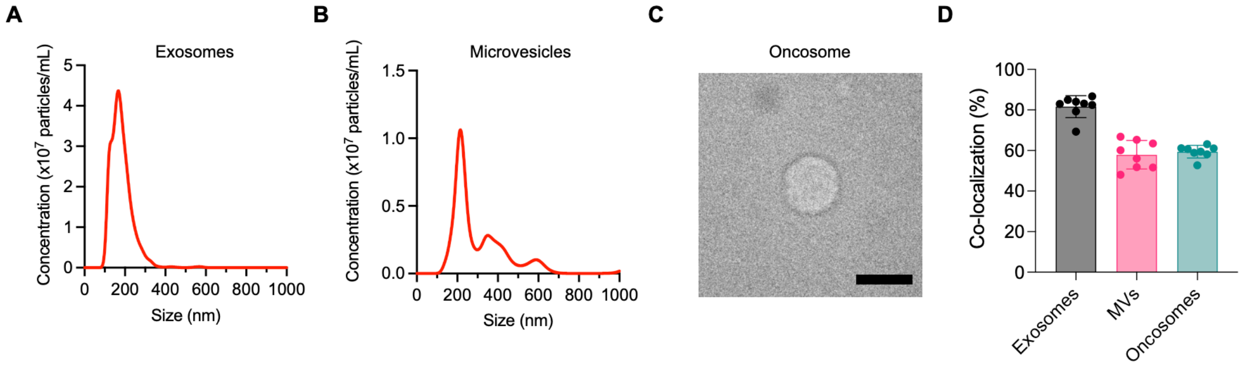

Figure S3. **A-B.** Size distribution of exosomes (A) and microvesicles (B) measured by nanoparticle tracking analysis. **C.** Representative microscopic image of oncosomes. Scale bar: 10 µm. **D**. Percentages of fusion efficiencies, as conducted by incubation with liposomes containing FAM-conjugated oligos.

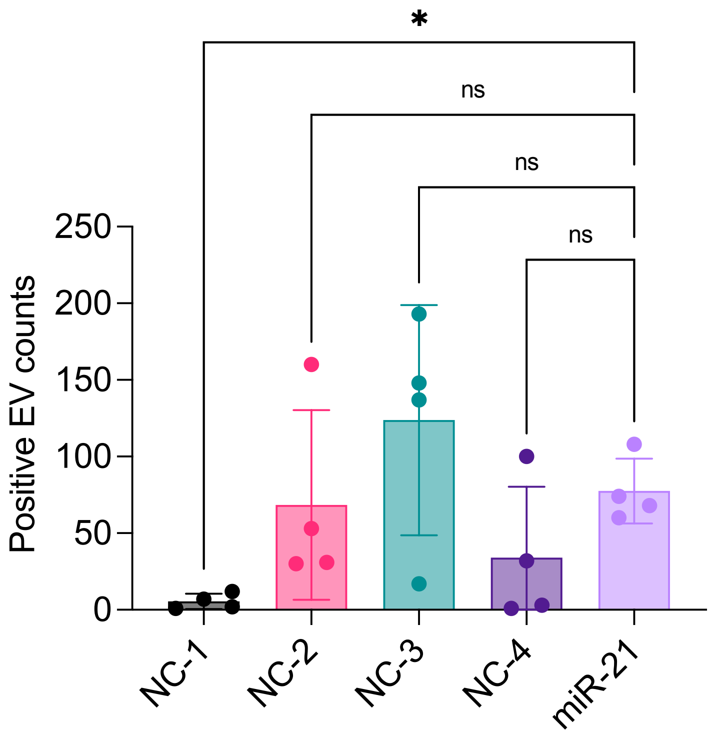

Figure S4. Selection of negative control (NC) crRNA. Four different sequences of NC crRNA were constructed by *in vitro* transcription and compared targeting efficiency with miR-21-5p crRNA. ns, not significant; *$P < 0.05$ compared with miR-21 crRNA samples, as assessed by two-way ANOVA with Bonferroni's multiple comparisons tests.

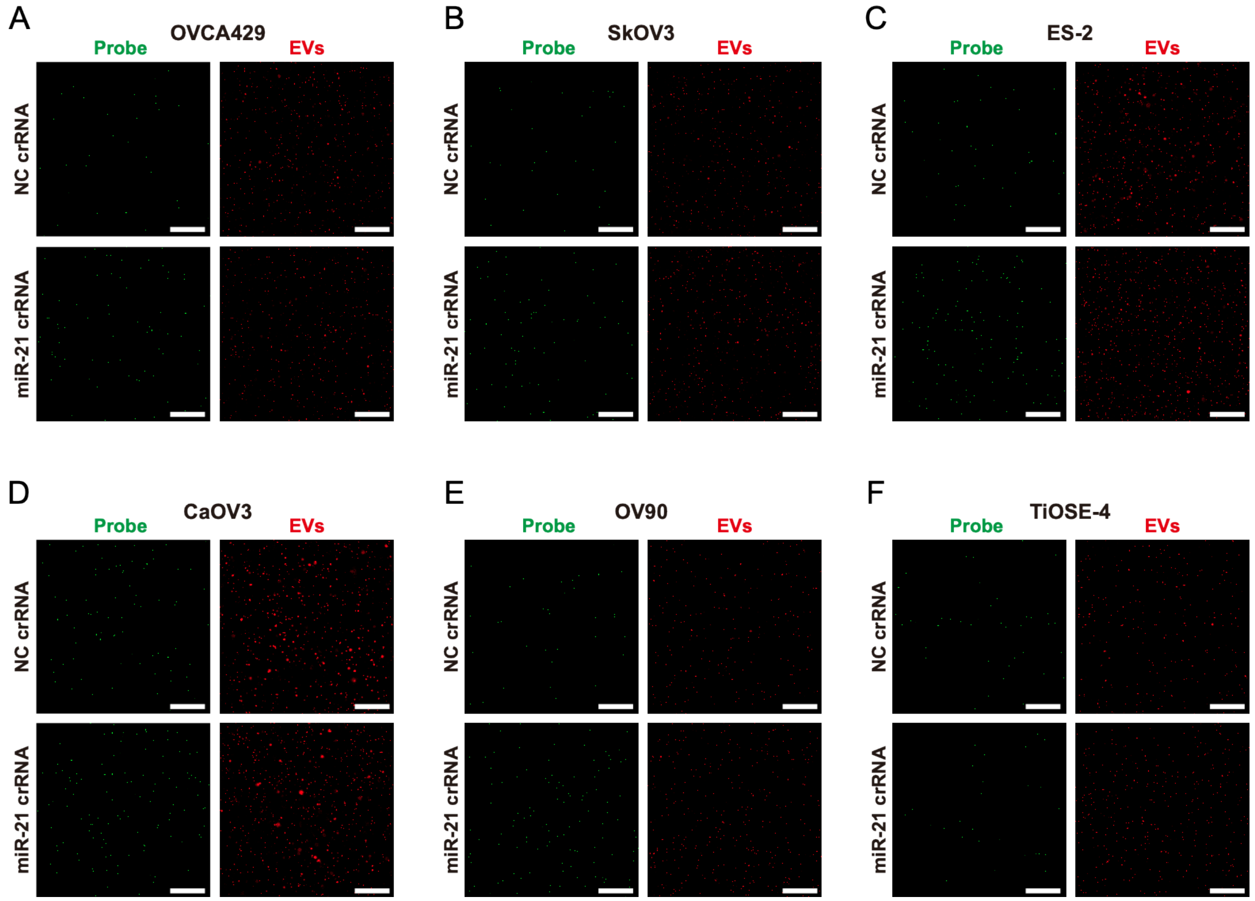

Figure S5. Representative fluorescence images of the EV miRNA detection assay in EVs from 5 different ovarian cancer cell lines and benign cell line. The miR-21-5p signals (left) and fluorescently labeled EVs (right) from OVCA429 **(A)**, SkOV3 **(B)**, ES-2 **(C)**, CaOV3 **(D)**, OV90 **(E)**, and TiOSE4 **(F)** are shown. Scale bar, 150 μm.

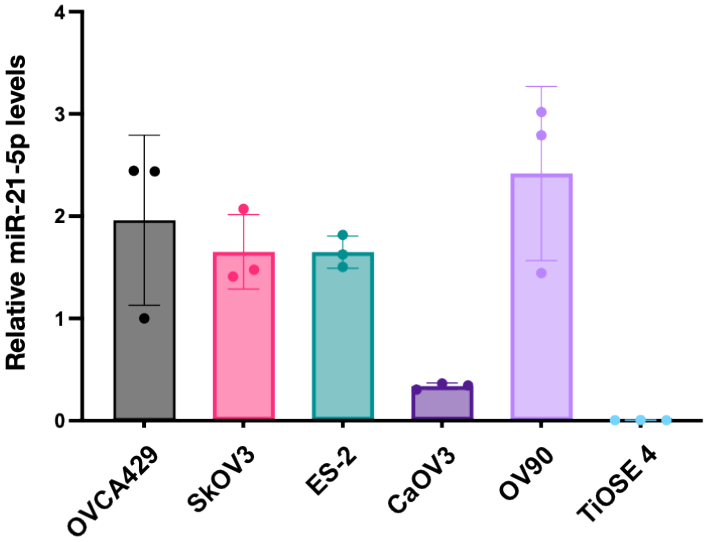

Figure S6. Relative abundances of miR-21-5p, as analyzed by RT-qPCR. U6 snRNA levels were served as an internal control. Bar graphs are shown as mean ± SD from the three independent experiments.

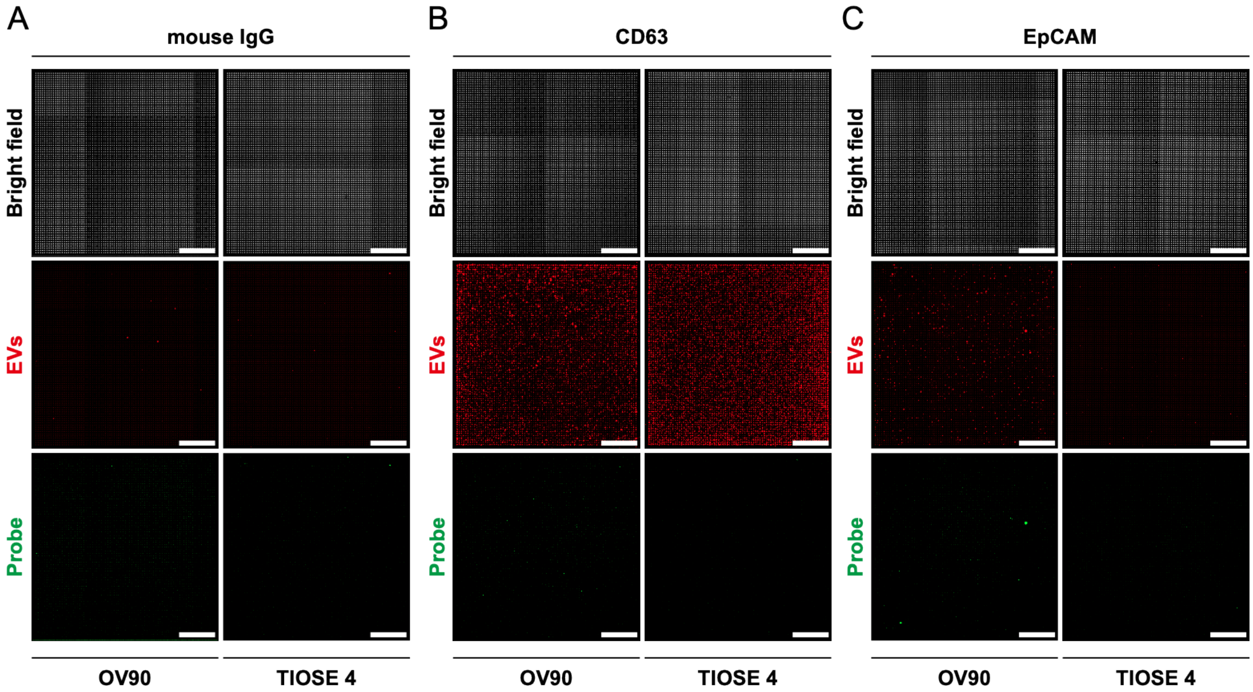

Figure S7. Detection of miRNA signature in EVs on antibody-immobilized gold micropattern. A-C. The OV90- or TiOSE4-derived EVs were immobilized by IgG isotope control **(A),** CD63 **(B), and EpCAM (C) antibody**. Representative images of gold micropattern in bright field (*upper*) as well as captured fluorescently labeled EVs (*middle)* and miR-21-5p signal (*bottom*) are shown. Scale bar, 150 μm.

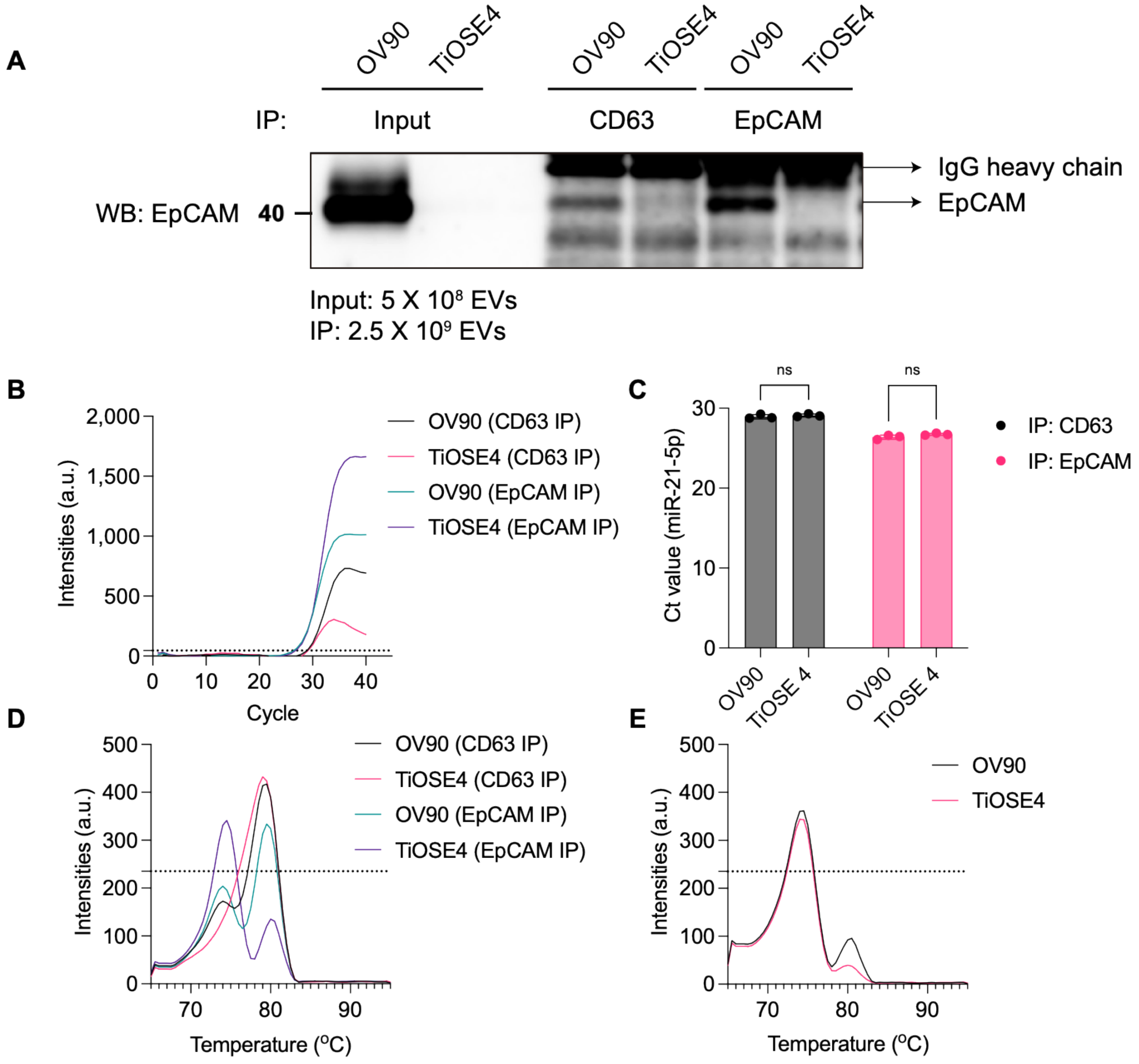

Figure S8. Detection of miR-21-5p levels in EpCAM-immunoprecipitated EVs by RT-qPCR. **A.** Validation of EV immunoprecipitation with CD63 and EpCAM antibodies, as conducted by western blot analysis. B-C. Amplification curves (B) and Ct value (C) of miR-21-5p, as performed by RT-qPCR. ns, not significant compared between EVs from OV90 and TiOSE4 cell lines, as assessed by two-way ANOVA with Bonferroni's multiple comparisons tests. Bar graphs are shown as mean ± SD from the three independent experiments. D-E. Melting curve analysis for the PCR products from CD63- or EpCAM-immunoprecipitated EVs (D) and intact EVs (E). Dotted lines indicate the intensity threshold value to determine the Ct value or melting peak.

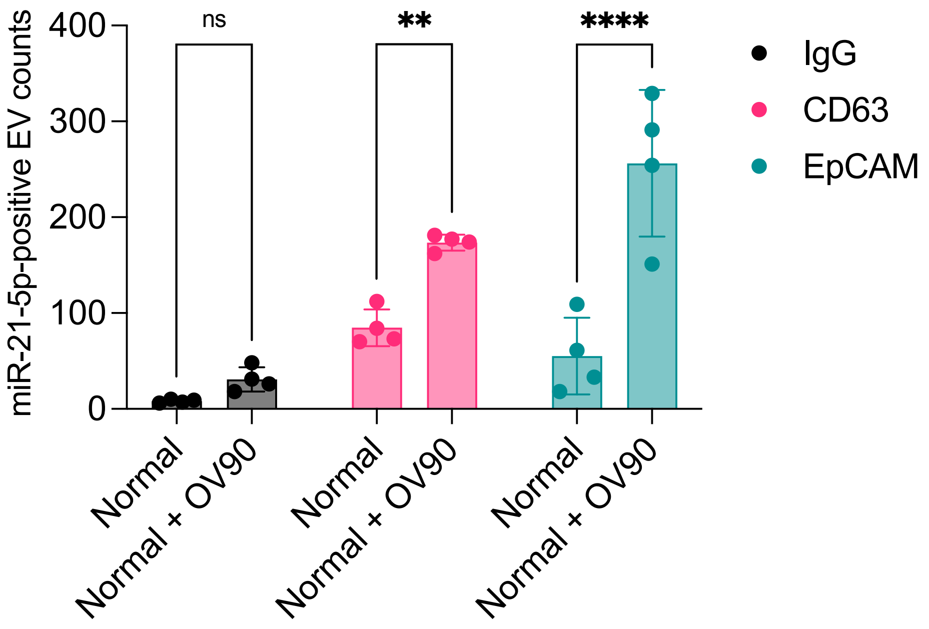

Figure S9. Detection of miR-21-5p in a plasma sample. The numbers of miR-21-5p-positive EVs in IgG-, CD63- or EpCAM-positive EV subpopulations from healthy donor plasma and OV90 EV spiked-in samples. All bar graphs are shown as mean ± SD. **P < 0.01, ****P < 0.0001 compared with the Normal and Normal + OV90, as assessed by two-way ANOVA with Bonferroni's multiple comparisons tests.

Supplemental Table S1. Sequences of oligomers used in this study.

Name	Sequence (5' to 3')[ab]
**DNA**	
miR-21-5p-F	TAGCTTATCAGACTGATGTTGA
U6 snRNA-F	CAAATTCGTGAAGCGTTC CA
universal-R	AGGCAGTGGTATCAACGCAGA
miR-RT-adaptor	AGGCAGTGGTATCAACGCAGAGTACTTTTTTTTTTTTTTTTTTTTTTTTTTTTTTTTVN
T7-univeral upper	TAATACGACTCACTATAG
T7-bottom NC-1	TTGTACTACACAAAGTACTGGTTTTAGTCCCCTTCATTTTTGGGGTGGTCTAAATCTATAGTGAGTCGTATTA
T7-bottom NC-2	TTGTACTACACAAAAGTACTGGTTTTAGTCCCCTTCATTTTTGGGGTGGTCTAAATCTATAGTGAGTCGTATTA
T7-bottom NC-3	CCCCCCCCCCCCCCCCCCCCCGTTTTAGTCCCCTTCATTTTTGGGGTGGTCTAAATCTATAGTGAGTCGTATTA
T7-bottom NC-4	AAAAAAAAAAAAAAAAAAAAAGTTTTAGTCCCCTTCATTTTTGGGGTGGTCTAAATCTATAGTGAGTCGTATTA
T7-bottom miR-21-5p	TAGCTTATCAGACTGATGTTGAGTTTTAGTCCCCTTCATTTTTGGGGTGGTCTAAATCTATAGTGAGTCGTATTA
**RNA**	
Synthtic miR-21-5p	phospho-uagcuuaucagacugauguuga
Syntetic NC-1	phospho-uuguacuacacaaaguacug
Poly U probe	FAM-uuuuu-Quencher
DRD probe	FAM-TAuuGC-Quencher
Double quencher probe	FAM-uuuuuuuu-Quencher-uu-Quencher

[a] V = A, C, or G; N = A, C, G, or T

[b] Lower case letters denote RNA

Supplemental Table S2. Clinical information of patients

Characteristic	Ovarian/Mullerian cancer (*n* = 5)	Healthy volunteers* (*n* = 3)
**Age, yr**		
Mean (range)	59 (52-63)	26.0 (19-33)
**Sex (%)**		
Female	5 (100)	3 (100)
**Stage of tumor (%)**		
I	0	-
II	0	-
III	0	-
IV	100	-
**Race/Ethnicity**		
Asian	0	0 (0)
Black	0	1 (33.3)
White	4 (80)	2 (66.7)
Other	1 (20)	0 (0)
Unknown	0	0 (0)

** Healthy volunteer samples were randomly chosen and provided by the MGB Biobank*

Supplemental Table S3. Comparison of EV-miRNA detection methods.

Method	LOD	Target miRNA	Principal sensing mechanism	EV lysis free	RNA extraction free	Target amplification free	Single EV analysis	Multiplexed (Protein-RNA) analysis	References
Ultra-sensitive electrochemical sensing	2.75 fM	miR-21	Electrochemistry	No	No	No	No	No	34
One-pot EXTRA-CRISPR miRNA assay	1.64 fM	miR-21	Cas12a/ cis- and trans-cleavage	No	No	No	No	No	35
DNA cube-based 3D nanomachine	77.4 pM	miR-21	Catalytic hairpin assembly	No	No	Yes	No	No	36
Dual-cycliing nanoprobe	66 fM	miR-21	Toehold switch	No	No	Yes	No	Yes	37
Rolling circle amplification within encoded hydrogel particles	2.3 zM<br>17 zM<br>44 zM	miR-21<br>miR-19b<br>let-7	Rolling circle amplification	No	Yes	No	No	No	38
All in one biosensor	0.116 μg/mL<br>0.125 μg/mL<br>0.287 μg/mL	miR-21<br>miR-27a<br>miR-375	Hybridization	Yes	Yes	Yes	No	No	39
MFS-CRISPR	1.2×10^3 particles/mL	miR-21-5p	Cas13a / trans-cleavage	Yes	Yes	Yes	No	No	29
Nanoflare	0.36 fM	miR-375	Thermophoretic accumulation	Yes	Yes	Yes	No	No	40
3D Microfluidic Chip	14 exosomes/μL<br>22 exosomes/μL<br>20 exosomes/μL	miR-451a<br>miR-21<br>miR-10b	Molecular beacon	Yes	Yes	Yes	No	Yes	41
Exo-PROS	1.2×10^3 exosomes/mL	EGFR+<br>miR-21	Molecular beacon	Yes	Yes	Yes	No	Yes	42
MB-CCP	N/A	miR-451a	Molecular beacon	Yes	Yes	Yes	Yes	No	43
This study	1×10^8 EVs<br>0.14% (LOQ)*	miR-21-5p	Cas13a / trans-cleavage	Yes	Yes	Yes	Yes	Yes	

*LOQ: limit of quantification
